# Improving access to affordable quality-assured inhaled medicines in low- and middle-income countries

**DOI:** 10.5588/ijtld.22.0270

**Published:** 2022-11-01

**Authors:** M. Stolbrink, M. J. Chinouya, S. Jayasooriya, R. Nightingale, L. Evans-Hill, K. Allan, H. Allen, J. Balen, T. Beacon, K. Bissell, J. Chakaya, C-Y. Chiang, M. Cohen, G. Devereux, A. El Sony, D. M. G. Halpin, J. R. Hurst, C. Kiprop, A. Lawson, C. Macé, A. Makhanu, P. Makokha, R. Masekela, H. Meme, E. M. Khoo, R. Nantanda, S. Pasternak, C. Perrin, H. Reddel, S. Rylance, P. Schweikert, C. Were, S. Williams, T. Winders, A. Yorgancioglu, G. B. Marks, K. Mortimer

**Affiliations:** 1 Clinical Sciences, Liverpool School of Tropical Medicine, Liverpool, UK; 2 Stellenbosch University, Tygerberg, South Africa; 3 Education Department, Liverpool School of Tropical Medicine, Liverpool, UK; 4 Academic Unit of Primary Care, University of Sheffield, Sheffield, UK; 5 IcFEM Dreamland Mission Hospital, Kimilili, Kenya; 6 Nifty Fox Creative, Sheffield, UK; 7 Healthcare Consultant, Medical Research Council Unit The Gambia at London School of Hygiene & Tropical Medicine, The Gambia; 8 Medical Research Council Unit The Gambia at London School of Hygiene & Tropical Medicine, The Gambia; 9 School of Health and Related Research, University of Sheffield, Sheffield, UK; 10 Medical Aid International, Bedford, UK; 11 School of Population Health, University of Auckland, Auckland, New Zealand; 12 Department of Medicine, Therapeutics and Dermatology, Kenyatta University, Nairobi, Kenya; 13 International Union Against Tuberculosis and Lung Disease, Paris, France; 14 Division of Pulmonary Medicine, Department of Internal Medicine, Wan Fang Hospital, Taipei Medical University, Taipei, Taiwan; 15 Division of Pulmonary Medicine, Department of Internal Medicine, School of Medicine, College of Medicine, Taipei Medical University, Taipei, Taiwan; 16 Asociación Latinoamericana del Tórax, Forum of International Respiratory Societies, Guatemala; 17 The Epidemiological Laboratory (Epi-Lab) for Public Health, Research and Development, Khartoum Sudan; 18 University of Exeter Medical School, College of Medicine and Health, University of Exeter, Exeter, UK; 19 UCL Respiratory, University College London, London, UK; 20 AstraZeneca, Cambridge, UK; 21 Department of Paediatrics and Child Health, School of Clinical Medicine, University of KwaZulu Natal, Durban, South Africa; 22 Centre for Respiratory Diseases Research, Kenya Medical Research Institute, Nairobi, Kenya; 23 Department of Primary Care Medicine, Faculty of Medicine, University of Malaya, Kuala Lumpur, Malaysia; 24 International Primary Care Respiratory Group, Larbert, Scotland, UK; 25 Makerere University Lung Institute, College of Health Sciences, Makerere University, Kampala, Uganda; 26 GlaxoSmithKline, Brentford, UK; 27 The Woolcock Institute of Medical Research, The University of Sydney, Sydney, NSW, Australia; 28 Global Initiative for Asthma (GINA), Fontana, WI, USA; 29 Noncommunicable Diseases Department, World Health Organization, Geneva, Switzerland; 30 Global Allergy & Airways Patient Platform, Vienna, Austria; 31 Department of Pulmonology, Celal Bayar University Medical Faculty, Manisa, Turkey; 32 Global Alliance Against Chronic Respiratory Diseases, Geneva, Switzerland; 33 University of New South Wales, Sydney, NSW, Australia; 34 University of Cambridge, Cambridge, UK

**Keywords:** asthma, COPD, non-communicable disease, chronic respiratory disease, inhalers, essential medicines

## Abstract

**BACKGROUND:**

Access to affordable inhaled medicines for chronic respiratory diseases (CRDs) is severely limited in low- and middle-income countries (LMICs), causing avoidable morbidity and mortality. The International Union Against Tuberculosis and Lung Disease convened a stakeholder meeting on this topic in February 2022.

**METHODS:**

Focused group discussions were informed by literature and presentations summarising experiences of obtaining inhaled medicines in LMICs. The virtual meeting was moderated using a topic guide around barriers and solutions to improve access. The thematic framework approach was used for analysis.

**RESULTS:**

A total of 58 key stakeholders, including patients, healthcare practitioners, members of national and international organisations, industry and WHO representatives attended the meeting. There were 20 pre-meeting material submissions. The main barriers identified were 1) low awareness of CRDs; 2) limited data on CRD burden and treatments in LMICs; 3) ineffective procurement and distribution networks; and 4) poor communication of the needs of people with CRDs. Solutions discussed were 1) generation of data to inform policy and practice; 2) capacity building; 3) improved procurement mechanisms; 4) strengthened advocacy practices; and 5) a World Health Assembly Resolution.

**CONCLUSION:**

There are opportunities to achieve improved access to affordable, quality-assured inhaled medicines in LMICs through coordinated, multi-stakeholder, collaborative efforts.

## Abstract (fr)

**CONTEXTE:**

Dans les pays à faible revenu et à revenu intermédiaire (LMIC), l’accès aux médicaments par inhalation abordables pour les maladies respiratoires chroniques (CRD) est extrêmement limité et entraîne une morbidité et une mortalité qui pourraient être évitées. L’Union internationale contre la tuberculose et les maladies respiratoires a réuni les parties prenantes concernées lors d’une réunion à ce sujet en février 2022.

**MÉTHODES:**

Les discussions ciblées de groupe étaient étayées par les données de la littérature et des présentations synthétisant les différentes expériences en matière d’accès aux médicaments par inhalation dans les LMIC. Le déroulement de la réunion en ligne respectait les thèmes définis dans un guide dédié portant sur les obstacles et solutions visant à améliorer cet accès. Cette approche thématique a été utilisée pour l’analyse.

**RÉSULTATS:**

Au total, 58 parties prenantes clés (dont patients, personnel soignant, membres d’organisations nationales et internationales, représentants de l’industrie et de l’OMS) ont pris part à cette réunion. Avant la réunion, 20 éléments de documentation ont été soumis. Les principaux obstacles identifiés étaient : 1) une mauvaise connaissance des CRD, 2) des données limitées sur le poids sanitaire des CRD et les traitements dans les LMIC, 3) l’inefficacité des réseaux d’approvisionnement et de distribution et 4) une mauvaise communication quant aux besoins des patients atteints de CRD. Les solutions évoquées étaient : 1) génération de données pour orienter les politiques et la pratique, 2) renforcement des capacités, 3) amélioration des mécanismes d’approvisionnement, 4) renforcement des pratiques de plaidoyer et 5) adoption d’une résolution par l’Assemblée mondiale de la santé.

**CONCLUSION:**

Il est possible d’améliorer l’accès à des traitements par inhalation abordables de qualité dans les LMIC, au travers d’efforts collaboratifs et coordonnés impliquant toutes les parties prenantes.

The rising burden of non-communicable diseases (NCDs) represents a substantial threat to health and development worldwide; 41 million people each year die of an NCD, and the burden of NCDs affects the poorer segments of society the most; 77% of all NCD deaths occur in low- and middle-income countries (LMICs).^1^ The prevention and control of NCDs, including asthma and chronic obstructive pulmonary disease (COPD), is a global health priority highlighted by UN General Assembly High-Level Meetings in 2011, 2014 and 2018, and the UN High-Level Meeting on Universal Health Coverage (UHC) in 2019.^2^ A disproportionately high burden of symptoms, disability and death from chronic respiratory diseases (CRDs) occurs in LMICs. Access to affordable, safe and effective medicines is central to NCD management, and reflected in targets from the WHO Global Action Plan For The Prevention and Control of Noncommunicable Diseases and the 2030 Agenda for Sustainable Development Goals.^3^,^4^ Inhaled medicines are included in the WHO Model List of Essential Medicines and should therefore be “available at all times in adequate amounts”.^5^ In practice, inhalers are often not available or unaffordable in LMICs.^6^–^8^

Previous efforts by the International Union Against Tuberculosis and Lung Diseases (The Union) to facilitate procurement of quality-assured, essential, affordable asthma medications for LMICs, the Asthma Drug Facility Programme, were discontinued in 2013 due to factors, including lack of political commitment and limited local disease management strategies.^9^,^10^ Multiple agencies and stakeholders influence the reliable access to inhaled medicines in LMICs.^11^ The Union convened a meeting on this topic in February 2022. The objectives were to assemble global stakeholders with widespread perspectives to discuss barriers to access to affordable, quality-assured inhaled medicines for people with CRDs (mainly asthma and COPD) in LMICs, explore possible solutions and suggest priorities for future work.

## METHODS

The initiative was led by GM and KM (on behalf of The Union), who identified key stakeholders drawing on The Union’s extensive networks. Efforts were made to be inclusive of a broad range of stakeholders to obtain viewpoints from various backgrounds.

These key stakeholders were engaged in procurement, treatment and policy-making decisions relating to the use of inhaled medicines in LMICs. They included patients, academics, representatives of international multilateral organisations, pharmaceutical companies and clinicians, and are included as authors of this paper. They were individually approached from July 2021 and invited to participate in the virtual group discussion. All contributors provided consent. No formal ethics approval was required. The preparation of, and the meeting itself, was undertaken during the COVID-19 pandemic which affected the ability of some to participate and necessitated a virtual conversation. In preparation for the meeting, contributors submitted audio-recorded presentations on barriers, potential solutions and pathways to the solutions from their perspective. These, and two recently published articles, were available to all contributors in advance to inform the discussions.^8^,^12^

The 2-hour virtual meeting was hosted, recorded and transcribed on Zoom (zoom.us) on 4 February 2022. During the discussions, participants were able to post comments and questions on the chat facility. The focus group discussions involved structured conversations moderated by facilitators (MS, MC, RN, SJ), who used a topic guide designed around “barriers to access”, “solutions” and “pathway to solutions” (Supplementary Table S1). The facilitators developed the topic guide in collaboration with GM and KM, in line with strategic objectives of The Union and the WHO. Key messages emerging from the discussions were captured by a visual storyteller (LEH). LEH created visual imagery outputs in real-time that were used iteratively by the facilitators to engage the participants.^13^

The transcript of the meeting, the chat and the visual imageries were analysed using the thematic framework approach with NVivo v12 (QSR International, Warrington, UK; 2018). This is a qualitative approach involving the analysis of key patterns in meaning and the identification of broad themes from qualitative data.

## RESULTS

**Figure 1:**
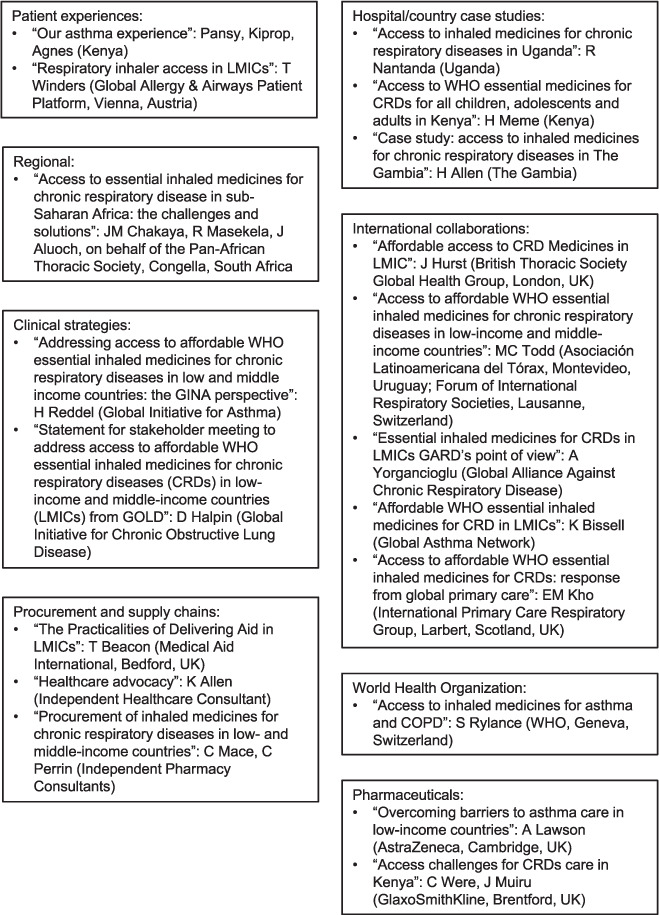
Overview of the presentations available pre-meeting. (https://prezi.com/view/eOuY7719eZlGKvrgf1Vj/). LMIC = low- and middle-income countries; GINA = Global Initiative for Asthma; CRD = chronic respiratory disorder; GOLD = Global Initiative for Chronic Obstructive Lung Disease; GARD = Global Alliance Against Chronic Respiratory Disease; COPD = chronic obstructive pulmonary disease.

Twenty stakeholders provided presentations that were available pre-meeting and 58 people attended the meeting (Figure 1, Supplementary Table S2, https://prezi.com/view/eOuY7719eZlGKvrgf1Vj/). The following themes emerged.

### Barriers

**Figure 2:**
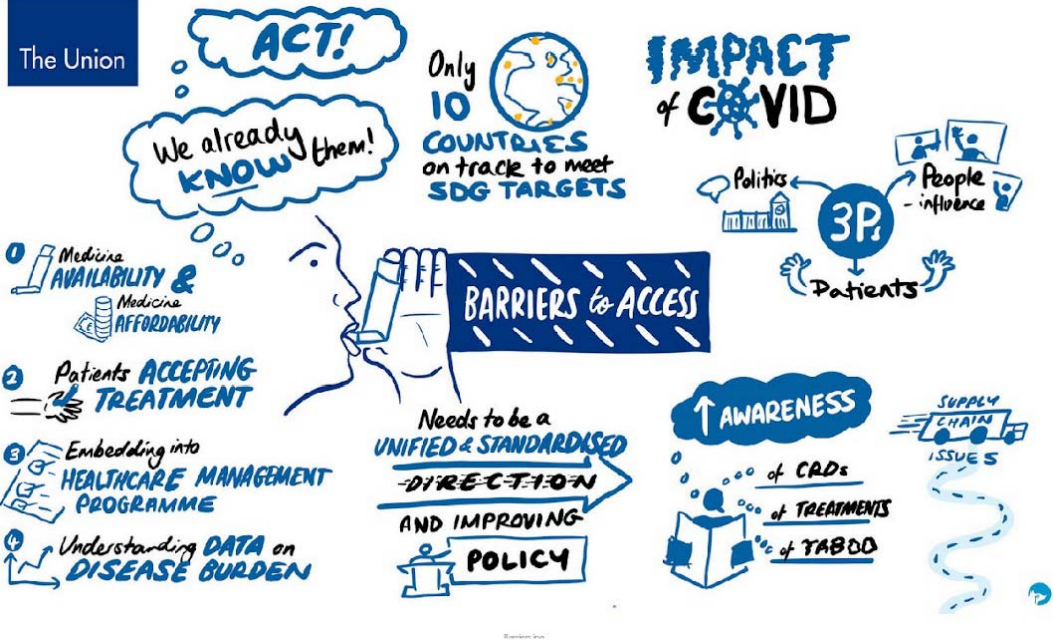
Visualisation created during the meeting: discussion of barriers. SDG = Sustainable Development Goals; CRD = chronic respiratory disorder.

There was consensus among contributors that the barriers to the access of affordable inhaled medicines in LMICs were multi-faceted, complex, and involved many actors (Figure 2).

### Low awareness of CRDs

Low awareness of CRDs and their treatment among a spectrum of stakeholders was identified as a key barrier. The lack of community knowledge about diagnosis, treatment and outcomes for CRDs and the resultant stigma surrounding the use of inhaled medications was highlighted. A lack of awareness of evidence-based diagnostics and treatments among healthcare providers, including pharmacists and other allied healthcare workers, was discussed. This lack of awareness was closely related to the paucity of local and LMIC-specific guidelines, limited availability of medicines, medical equipment and shortcomings in training. The lack of awareness of the impact of CRDs by local policymakers, healthcare planners, governments and global organisations was thought to be driven by sparse and poorly understood data on disease burden and competing priorities, such as communicable diseases and ‘competing’ NCDs such as diabetes and hypertension. Many LMIC practitioners work within fragmented healthcare systems with multi-layered, bureaucratic decision-making that delay execution of the latest management strategies and guidelines.

Inhaled medicines can be complex products to use correctly. Sub-optimal use of ‘controller’ and overreliance on ‘reliever’ inhalers are important factors for the lack of control of CRD symptoms, and for excess mortality and morbidity. Limited patient and healthcare provider training and knowledge on the use of the vast variety of inhaler devices were highlighted, further challenged by recurrent supply issues, precipitating frequent device and formulation changes. The use of outdated, oral agents in preference to inhaled medications was also recognised. High prices and unreliable supplies of medicines were quoted by patients as being a barrier to receiving effective treatment.

### Limited data on CRD burden and treatment

Limited data on local disease burden, economic impact, and ultimately, medication demand, in combination with inefficient and unpredictable procurement mechanisms and supply chains, create challenges to industry and governments in providing reliable access to inhaled medicines. CRD care was often not fully integrated into the health system, making training, monitoring and infrastructure development difficult. The negative impact of the COVID-19 pandemic on health systems in LMICs was discussed, as was governments having multiple competing interests but limited resources. Challenging local regulations, unsuitable incentives to register products and weak regulatory systems for medicines may result in inappropriate treatments being available.

### Ineffective procurement and distribution networks

Disparities within and between countries were discussed as a barrier. For example, access to medicines and diagnostics differed dramatically between tertiary care, urban areas and private hospitals compared to primary care and rural health posts. Most of the population in rural or peri-urban settings is served by primary healthcare, yet much of the current knowledge about CRDs in LMICs derives from cities and hospitals. Different countries have diverse health systems, priorities and guidelines, making it difficult for the industry and others to predict demand for medicines. The lack of demand does not correspond to a lack of need. The lack of standardised national protocols and treatment algorithms for CRDs incentivises market fragmentation, resulting in poor visibility of opportunities for manufacturers, potentially resulting in irregular supply. Receiving regulatory approval for a medicine can be a lengthy complicated process that differs from country to country. Without well-defined, consistent demand and strong CRD policies, the industry may be reluctant to invest in registering products and supplying them.

### Poor communication of the needs of people with CRDs

Ineffective communication by a range of stakeholders at the local, national, regional and global levels was highlighted as a significant barrier to obtaining funding, and hence, access to medicines. Limited advocacy by patients, professionals and respiratory organisations was considered a barrier to informing and influencing local and global policymakers and funders (Figure 2).

### Solutions and pathways to solutions

#### Generation of data to inform policy and practice

**Figure 3:**
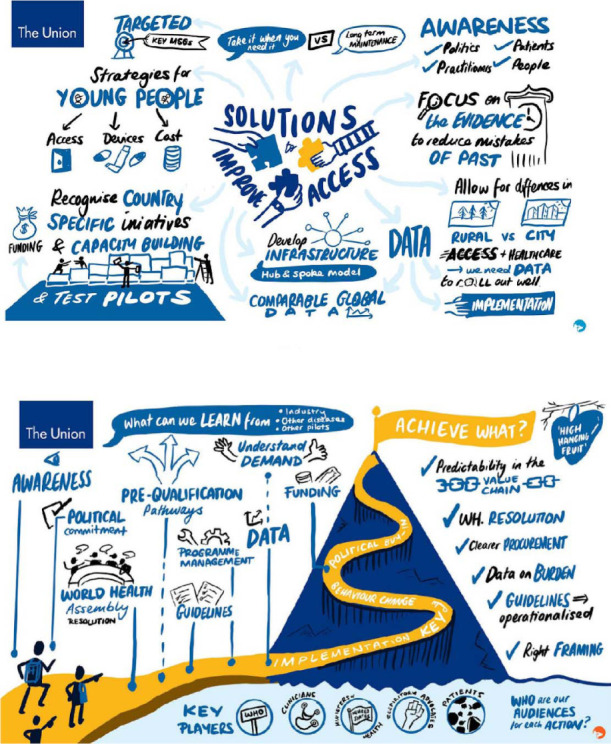
Visualisations created during the meeting: discussions about solutions.

Sharing of knowledge was identified as key to improving access to inhaled medicines (Figure 3). This could involve learning from successful projects in specific settings. Also discussed was the possibility of future work being focused on programmatic and implementation projects addressing rural and lower health tier centres to understand what is scalable and replicable to other healthcare setting and contexts. Global organisations, academic institutions and industry would be key in providing the infrastructure for this. Updated guidance for LMICs, e.g., the WHO package of essential NCD (PEN) interventions for primary health care, could be used, although new evidence about more effective and safer strategies than those used in the past should be adopted. Lessons from previous projects, such as the Asthma Drug Facility, should be shared. There also needs to be learning from recent promising initiatives in relation to other NCDs, such as diabetes.

### Capacity building

Sharing of knowledge locally, such as enhancing capacity and capability of healthcare professionals in more remote areas and improving health literacy of the communities, was examined. Communities working closely with healthcare workers and pharmacists on addressing misconceptions and providing training to use inhaled medicines appropriately would improve care. The implementation of pragmatic treatment guidelines, programmes and essential medicines lists will be required. Suggestions were made to integrate CRD care into already established local healthcare programmes and into efforts to roll out UHC, which would also benefit other NCDs. UHC might also offer diagnostic equipment, knowledge exchange and capture data on the burden of disease, costs, individual needs and the healthcare system, which will ultimately aid advocacy.

### Improved procurement

Sharing knowledge on successful procurement programmes and transparent pricing of medicines to improve buying power from individual countries was also discussed. Using standardised methodologies or sharing resources to conduct these projects in LMICs would allow easier comparisons and benchmarking of prices. Collecting data to facilitate demand prediction would be key to providing stability for the supply chain.

As inhaled formulations are complex to assess from a regulatory standpoint, expanding the scope of WHO pre-qualification to inhalers may aid with local approvals. Procurement mechanisms will be improved by establishing national treatment guidelines in line with international guidance and using real competition between quality-assured innovator and generic inhaled formulations when procuring medicines.

### Strengthening advocacy

Patient national and international advocacy groups together with WHO advocacy will be key in influencing governments’ agendas. Effective, targeted communication among politicians, patients, practitioners and the general population will be essential for this. The vital role of patients was highlighted through recent successes, such as the World Health Assembly (WHA) Resolution for Diabetes, which were strongly supported by patient representation.

### World Health Assembly Resolution

Ultimately, political commitments by individual countries for funding, policies and investment are needed. A WHA Resolution on care for all children, adolescents and adults with CRDs would help focus attention and resources on implementing the solutions needed. Close collaboration between the WHO, clinicians, ministries of health, respiratory advocates, communities and patients will be needed to achieve such a resolution and implement its recommendations (Figure 3).

## DISCUSSION

**Figure 4:**
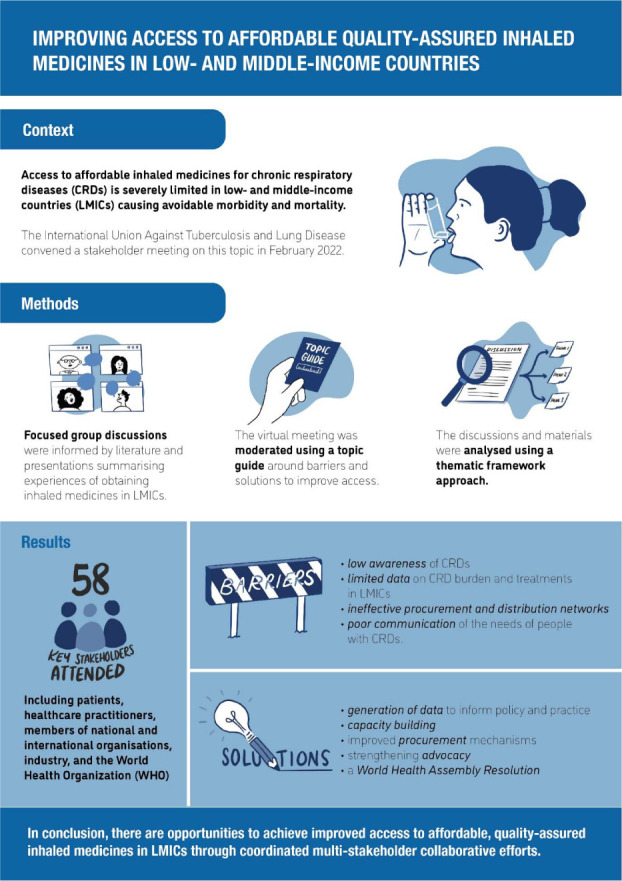
Visual abstract.

Several barriers to accessible, affordable and quality-assured inhaled medicines in LMICs were identified, including limited awareness, paucity of data, ineffective procurement and poor communication (Figure 4). Many obstacles could be overcome through investment in collaborative stakeholder solutions. The five main solutions identified were generation of data to inform policy and practice; capacity strengthening; improved procurement mechanisms and distribution networks; improved advocacy; and a WHA Resolution on CRDs focused on access to affordable quality-assured effective care for all children, adolescents and adults who would benefit from them. Patients, governments, communities, the industry and practitioners will be instrumental in bringing these to fruition (Figure 4).

The use of standardised tools, such as an updated WHO PEN guideline, the WHO/Health Action International (London, UK) or MedMon methodologies, allow reliable data collection and benchmarking among countries.^14^–^16^ This may ultimately not only result in improved health but also strengthen advocacy and more efficient procurement mechanisms. For procurement, countries may take advantage of published guidance, such as WHO norms and standards for health products, and guidelines for authorisation, medicine prequalification, distribution and quality control.^17^–^19^ Implementation research will be especially important.^20^–^22^ Public and private partnerships to improve CRD care in LMICs are underway and will form pilots for this.^23^–^25^ The Asthma Drug Facility experience showed that such a programme succeeded at keeping prices affordable, but was limited by the absence of dedicated local budgets, updated national essential medicines lists and national strategies for asthma, and so was ultimately unable to scale up and secure permanent funding.^10^ The respiratory community could benefit from learning from the diabetes and HIV communities, whose empowering of patients to advocate made a large impact on access to medicines.^26^,^27^

The strengths of our approach included the participation of a wide range of relevant stakeholders, including patients, patient advocacy organisations, national and international respiratory societies, global organisations that develop CRD management strategies, pharmaceutical companies, the Global Alliance Against CRDs (GARD) and the WHO. The structured approach to the organisation and implementation of the meeting, drawing on qualitative research methodology, allowed us to take a robust approach to the analysis and synthesis of the discussions. Limitations included there being other stakeholders, outside of The Union’s networks, who were not contributors and could have brought valuable perspectives. Challenges included conducting a virtual meeting, the large number of participants and limited time for discussion.

To our knowledge, this is the first time an international meeting has been convened to tackle this topic since the World Asthma Meeting in 1998.^28^ Improvements to NCD treatments can be made, as demonstrated by the diabetes community and their success in improving access to insulin treatment by advocating for a WHA Resolution in 2021.^29^,^30^ A similar initiative for CRDs could bring the same benefits for children, adolescents and adults with CRDs in LMICs.

**Figure 5:**
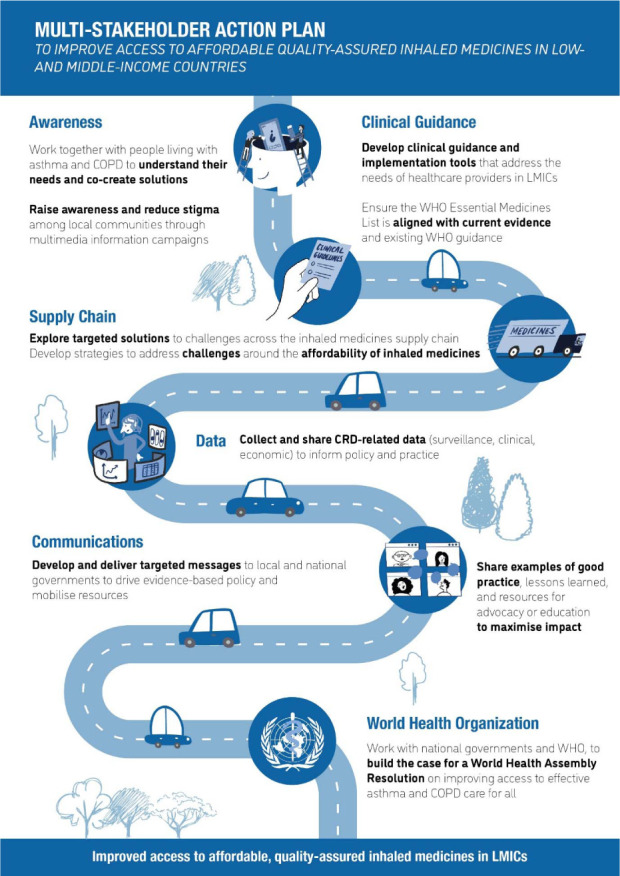
Multi-stakeholder action plan. COPD = chronic obstructive pulmonary disease; LMIC = low- and middle-income countries; CRD = chronic respiratory disorder.

In conclusion, we suggest that with sufficient targeted resources, collaborative multi-stakeholder action could overcome the barriers in access to affordable, quality-assured, essential inhaled therapies for people with CRDs in LMICs. Given the expertise and experience within this group of stakeholders, we suggest the following areas for collaborative action: 1) work together with people living with asthma and COPD to understand their needs and co-create solutions; 2) raise awareness and reduce stigma among local communities using multimedia information campaigns; 3) develop clinical guidance and implementation tools that address the needs of healthcare providers in LMICs; 4) ensure that the WHO Essential Medicines List is aligned with current evidence and existing WHO guidance; 5) explore targeted solutions to challenges across the inhaled medicines supply chain continuum, particularly to streamline procurement processes and to guarantee the quality of products; 6) develop strategies to address challenges around the affordability of inhaled medicines; 7) collect and share CRD-related data (surveillance, clinical, economic) to inform policy and practice; 8) develop and deliver targeted messages to local and national governments to drive evidence-based policy and mobilise resources for CRDs; 9) work with national governments and the WHO to build the case for a WHA Resolution on improving access to effective asthma and COPD care for all; and 10) share examples of good practice, lessons learnt and resources for advocacy or education to maximise impact (Figure 5).

## References

[1] World Health Organization (2021). WHO Factsheet. Noncommunicable diseases. https://www.who.int/news-room/fact-sheets/detail/noncommunicable-diseases.

[2] NCD Alliance (2022). Supporting prevention & control of NCDs. https://ncdalliance.org/.

[3] World Health Organization (2013). 2013–2020 Global Action Plan for the prevention and control of noncommunicable diseases. http://apps.who.int/iris/bitstream/10665/94384/1/9789241506236_eng.pdf.

[4] United Nations (2021). Sustainable Development Goals. https://sdgs.un.org/goals.

[5] World Health Organization (2021). WHO Model List of Essential Medicines - 22^nd^ List. https://www.who.int/leishmaniasis/burden/Leishmaniasis_India/en/.

[6] Babar ZUD (2013). The availability, pricing and affordability of three essential asthma medicines in 52 low- and middle-income countries. Pharmacoeconomics.

[7] Mendis S (2007). The availability and affordability of selected essential medicines for chronic diseases in six low- and middle-income countries. Bull. World Health Organ.

[8] Stolbrink M (2022). The availability, cost and affordability of essential medicines for asthma and COPD in low-income and middle-income countries: a systematic review. Lancet Global Health.

[9] Billo N (2006). Asthma drug facility: from concept to reality. Int J Tuberculosis Lung Dis.

[10] Chiang C-Y (2022). The Asthma Drug Facility and the future management of asthma. Int J Tuberc Lung Dis.

[11] Bissell K, Perrin C, Beran D (2016). Access to essential medicines to treat chronic respiratory disease in low-income countries. Int J Tuberc Lung Dis.

[12] Meghji J (2021). Improving lung health in low-income and middle-income countries: from challenges to solutions. Lancet.

[13] Bird K, Scharmer CO (2018). Generative scribing: a social art of the 21^st^ century.

[14] World Health Organization (2020). WHO package of essential noncommunicable (PEN) disease interventions for primary health care. WHO NCD Management Screening Diagnosis 1–77. https://www.who.int/publications/i/item/who-package-of-essential-noncommunicable-(pen)-disease-interventions-for-primary-health-care.

[15] World Health Organization & Health Action International (2008). Measuring medicine prices, availability, affordability and price components.

[16] World Health Organization (2018). MedMon - WHO essential medicines and health products price and availability monitoring mobile application. https://www.who.int/news/item/18-02-2018-medmonmobile-application.

[17] World Health Organization (2022). Guidelines: Norms and Standards for Pharmaceuticals. Health product policy and standards. https://www.who.int/teams/health-product-and-policy-standards/standards-and-specifications/norms-and-standards-for-pharmaceuticals/guidelines.

[18] World Health Organization (2022). WHO Global Benchmarking Tools for evaluation of national regulatory systems. https://www.who.int/tools/global-benchmarking-tools.

[19] World Health Organization (2022). Health product policy and standards. https://www.who.int/teams/health-product-policy-and-standards.

[20] Collins D (2019). Protocol for the evaluation of a pilot implementation of essential interventions for the prevention of cardiovascular diseases in primary healthcare in the Republic of Moldova. BMJ Open.

[21] Marten R (2021). Committing to implementation research for health systems to manage and control non-communicable diseases. Lancet Glob Health.

[22] Hurst JR (2021). Challenges in the implementation of chronic obstructive pulmonary disease guidelines in low- and middle-income countries: an official American Thoracic Society Workshop report. Ann Am Thorac Soc.

[23] AstraZeneca (2022). African Cluster: Pumua Initiative. https://www.astrazeneca.com/country-sites/african-cluster.html.

[24] Rockers PC (2019). Effect of Novartis access on availability and price of non-communicable disease medicines in Kenya: a cluster-randomised controlled trial. Lancet Glob Health.

[25] AMREF Health Africa (2018). Management and control of ncds (diabetes and childhood asthma) in Kenya. https://aidstream.org/files/documents/GSK-Amref-NCDs-Final-Narrative-Report_submission-file-20190214030242.pdf.

[26] Beran D, Hirsch IB, Yudkin JS (2018). Why are we failing to address the issue of access to insulin? A national and global perspective. Diabetes Care.

[27] Hoos A (2015). Partnering with patients in the development and lifecycle of medicines: a call for action. Ther Innov Regul Sci.

[28] Sterk PJ (1999). The message from the World Asthma Meeting. Eur Respir J.

[29] NCD Alliance (2021). WHA74 adopts landmark resolution on diabetes.

[30] World Health Organization (2021). The Global Diabetes Compact: what you need to know. https://cdn.who.int/media/docs/default-source/country-profiles/diabetes/gdc_need_to_know_web.pdf?sfvrsn=dddcb962_1&download=true.

