# Supplementary material for: Improving access to affordable quality-assured inhaled medicines in low- and middle-income countries
Source: Int J Tuberc Lung Dis. 2022 Nov 1;26(11):1023–32. doi: 10.5588/ijtld.22.0270 (PMC9621306; doi:10.5588/ijtld.22.0270)
Supplement: Supplementary file 1 [file iutld_ijtld_22.0270_supplementarydata1.pdf]

http://dx.doi.org/10.5588/ijtld.22.0270

SUPPLEMENTARY DATA

Improving access to affordable quality-assured inhaled medicines in low- and middle-income countries

Supplementary Table 1:	Topic guide to facilitate discussions
Supplementary Table 2:	List of organisations / institutions / representatives that provided pre-meeting materials

Supplementary Table 1: Topic guide to facilitate discussions

Main barriers to essential, affordable inhaled medication

Describe the main barriers in providing access to affordable, essential inhaled medications

- Share your thoughts of the submitted presentations and summaries – what issues did you detect?
- [Share your own experiences – what is your experience of access to inhaled medication?]
- Are there any other issues you have thought of after some more time and reflection? Are there any other gaps in access?
- Probe: financial; education; government interest; international interest; conflict; severity of symptoms / number of deaths; conflicting interests (e.g. HIV, TB) in health systems; patient perceptions and expectations; environmental; public health (tobacco)

Solutions to barriers

What do you think the solutions are to overcoming these barriers? What do you think would help overcome the barriers we have identified?

- Theoretical / visionary solutions versus realistic solutions
- What are the solutions that we can work on together as a community? (As opposed to national / local solution)

Pathways to solutions

How do we achieve these solutions?

- What do you think needs to be in place to achieve this?
- How could we work towards putting these in place?
- What are realistic ways of achieving them?
- Who needs to be involved?

Next steps

In your opinion, what are the next concrete steps?

- As a group of stakeholders
- When do we want these solutions to be achieved?
- Who should bring about these steps?

Supplementary Table 2: List of organisations / institutions / representatives that provided pre-meeting materials in alphabetical order

- Asociación Latinoamericana del Tórax (ALAT)
- AstraZeneca
- British Thoracic Society Global Health Group
- Forum of International Respiratory Societies (FIRS)
- Global Allergy & Airways Patient Platform (GAAPP)
- Global Alliance Against Chronic Respiratory Diseases (GARD)
- Global Asthma Network (GAN)
- Global Initiative for Asthma (GINA)
- Global Initiative for Chronic Obstructive Lung Disease (GOLD)
- GlaxoSmithKline
- Independent healthcare advocacy consultant
- Independent pharmacist consultants in global health
- International Primary Care Respiratory Group (IPCRG)
- Kenya Medical Research Institute (KEMRI)
- Medical Aid International
- Pan-African Thoracic Society (PATS)
- Case studies including patient voices:
  - Kenya
  - The Gambia
  - Uganda
- The International Union against Tuberculosis and Lung Disease (The Union)
- World Health Organization (WHO)
